# Supplementary material for: Knowledge of and preparedness for COVID-19 among Somali healthcare professionals: A cross-sectional study
Source: PLoS One. 2021 Nov 23;16(11):e0259981. doi: 10.1371/journal.pone.0259981 (PMC8610262; doi:10.1371/journal.pone.0259981)
Supplement: S2 File — (DOCX) [file pone.0259981.s002.docx]

Somalia HCP - v2

Start of Block: Introduction

Q0 Dear Participant, 
   You are invited to participate in this study, which aims to assess knowledge and perceptions surrounding the COVID-19 outbreak in Somalia among Somali health personnel. There will be no direct benefit to you as a participant in this study, but the results of the study may contribute to an improved response to the COVID-19 outbreak by the Somali government and non-governmental organizations. This survey was designed by Somalia’s Hargarla Institute, in consultation with colleagues from Yale University and the University of Cambridge.   

 Your participation in this study is voluntary and confidential and you will not need to write down your name.  Also, you can withdraw from it at any time. Your participation requires approximately ten minutes and requires that you fill out the all sections of the survey form. Filling out the survey confirms your consent to participate in this study. 
   If you have any questions, please contact us using the phone or email address provided below. 
   Dr. Deqo Mohamed, Founder and Director of the Hagarla InstituteEmail: waqafd36@gmail.com WhatsApp: +252 61 6691818

End of Block: Introduction

Start of Block: Demographics

Q1 How old are you?

- 18-24 (1)
- 25-34 (2)
- 35-44 (3)
- 45-64 (4)
- 65+ (5)

Q2 What is your gender?

- Male (1)
- Female (2)
- Other (Please Specify) (3) ________________________________________________

Q3 Which one of the following best describes you?

- Physician (1)
- Nurse (2)
- Midwife (3)
- Pharmacist (4)
- Dentist (5)
- Community Health Worker (6)
- Other Healthcare Professional (Please Specify) (7) ________________________________________________

Q4 Have you heard about COVID-19 (Coronavirus Disease 2019)?

- Yes (1)
- No (2)

Display This Question:

If Have you heard about COVID-19 (Coronavirus Disease 2019)? = Yes

Q5 When did you first hear about COVID-19?

- Before March 26th, 2020 (Date of first confirmed Somali case of COVID-19) (1)
- March 26th, 2020 or later (2)
- I'm Not Sure (3)

Display This Question:

If Have you heard about COVID-19 (Coronavirus Disease 2019)? = Yes

Q6 Have you received lectures or discussions about COVID-19?

- Yes (1)
- No (2)

Display This Question:

If Have you heard about COVID-19 (Coronavirus Disease 2019)? = Yes

Q7 Have you received information about how to best manage patients with COVID-19?

- Yes (1)
- No (2)

Display This Question:

If Have you heard about COVID-19 (Coronavirus Disease 2019)? = Yes

Q8 What sources do you use to gain information regarding COVID-19? [Choose all that apply]

- News, Media (e.g. TV, Radio, Newspapers, etc.) (1)
- Informational calls/SMS (2)
- Social Media (e.g. Facebook, Twitter, WhatsApp, YouTube, Instagram, Snapchat, etc.) (3)
- Official Government/International Websites (e.g. MoH, DoH, WHO, CDC, etc.) (4)
- Family Members, Colleagues, Friends (5)
- Employer, Work Colleagues, and Others at Work (6)
- Non-Governmental Organizations (NGOs) (7)
- Local or Community Leaders (8)
- Journals (9)
- Others (Please Specify) (10) ________________________________________________

Q9 On average, per week, how many patients did you see before March 26th (First confirmed Somali case of COVID-19)

- Patients (1) ________________________________________________

Q10 On average, per week, how has the number of patients that you have seen changed since the start of the pandemic, March 26th (First confirmed Somali case of COVID-19)

- It has increased and is now at _____ per week (1) ________________________________________________
- It has stayed the same at _____ per week (2) ________________________________________________
- It has decreased and is now at _____ per week (3) ________________________________________________

End of Block: Demographics

Start of Block: Knowledge of COVID-19

Display This Question:

If Have you heard about COVID-19 (Coronavirus Disease 2019)? = Yes

Q11 How is COVID-19 transmitted among humans? [Choose all that apply]

- Airborne Transmission (e.g. sneezing, coughing) (1)
- Direct contact of bodily fluids with infected persons (e.g. saliva) (2)
- Waterborne Transmission (3)
- Fecal-Oral Mode of Transmission (4)
- ⊗I don't know (5)

Display This Question:

If Have you heard about COVID-19 (Coronavirus Disease 2019)? = Yes

Q12 What is the incubation period of the Novel Coronavirus (SARS-CoV-2)?

- 2-7 days (1)
- 2-14 days (2)
- 7-14 days (3)
- 7-21 days (4)
- I don't know (5)

Display This Question:

If Have you heard about COVID-19 (Coronavirus Disease 2019)? = Yes

Q13 How would you evaluate the severity of COVID-19?

- Mild disease; always curable (1)
- Severe disease which can be fatal in certain cases (2)
- Always fatal (3)
- I don't know (4)

Display This Question:

If Have you heard about COVID-19 (Coronavirus Disease 2019)? = Yes

Q14 What percent of COVID-19 infections present as mild or asymptomatic?

- 20% (1)
- 45% (2)
- 55% (3)
- 80% (4)
- I don't know (5)

Display This Question:

If Have you heard about COVID-19 (Coronavirus Disease 2019)? = Yes

Q15 What are the symptoms of COVID-19? [Choose all that apply]

- Headache (1)
- Fever (2)
- Skin Rash (3)
- Dry Cough (4)
- Wet Cough or sputum/mucus production (5)
- Sore throat (6)
- Runny nose or nasal congestions (7)
- New loss of Taste and/or Smell (8)
- Shortness of breath or difficulty breathing (9)
- Diarrhea (10)
- Muscle or body aches (11)
- Fatigue or malaise (12)
- ⊗I don't know (13)

Display This Question:

If Have you heard about COVID-19 (Coronavirus Disease 2019)? = Yes

Q16 Which complications can arise in a patient with COVID-19? [Choose all that apply]

- Pneumonia (1)
- Respiratory Failure (2)
- Death (3)
- ⊗I don't know (4)

Display This Question:

If Have you heard about COVID-19 (Coronavirus Disease 2019)? = Yes

Q17 What is the current possible treatment of COVID-19?

- Supportive Care (1)
- Antiviral Therapy (2)
- Vaccination (3)
- I don't know (4)

Display This Question:

If Have you heard about COVID-19 (Coronavirus Disease 2019)? = Yes

Q18 How can one reduce the risk of transmission of COVID-19? [Choose all that apply]

- Hand hygiene (1)
- Covering nose and mouth when coughing (2)
- Freezing food that may be contaminted (3)
- Avoiding places where a large number of people are gathering (4)
- Avoiding sick contacts (5)
- ⊗I don't know (6)

End of Block: Knowledge of COVID-19

Start of Block: Experience + Attitudes

Display This Question:

If Have you heard about COVID-19 (Coronavirus Disease 2019)? = Yes

| 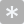 |
| --- |

Q19 Among the following symptoms, please indicate the five symptoms you see most frequently in suspected cases of COVID-19? [Only select "I have not seen any suspected cases of COVID-19" if that is the case]

- Headache (1)
- Fever (2)
- Skin Rash (3)
- Dry Cough (4)
- Wet Cough or sputum/mucus production (5)
- Sore throat (6)
- Runny nose or nasal congestions (7)
- New loss of Taste and/or Smell (8)
- Shortness of breath or difficulty breathing (9)
- Diarrhea (10)
- Muscle or body aches (11)
- Fatigue or malaise (12)
- I have not seen any suspected cases of COVID-19 (13)

Display This Question:

If Have you heard about COVID-19 (Coronavirus Disease 2019)? = Yes

Q20 Since March 26th (First confirmed Somali case of COVID-19), per week, how many patients do you see exhibiting COVID-19 symptoms

- Patients with symptoms (1) ________________________________________________

Display This Question:

If Have you heard about COVID-19 (Coronavirus Disease 2019)? = Yes

Q21 Since March 26th, on average per week, how many patients have you tested for COVID-19?

- Tests (1) ________________________________________________

Display This Question:

If Have you heard about COVID-19 (Coronavirus Disease 2019)? = Yes

Q22 Since March 26th, on average per week, how many patients with COVID-19 have you cared for or treated?

- Patients cared for or treated (1) ________________________________________________

Q23 Please rank the following in the order of which you believe are the most important to protect yourself against exposure while providing adequate care. (1 = "Most Important" and 7 = "Least Important")

______ Hand Sanitizer (1)

______ Disposable Gloves (2)

______ Disposable Gowns (3)

______ Disposable Masks (4)

______ N95 Masks (5)

______ Facial Protective Shields (6)

______ Telemedicine Capacities (7)

Q24 Please mark how easy or difficult it would be to acquire the following items.

|  | Very Hard (1) | Somewhat hard (2) | Not Sure (3) | Somewhat Easy (4) | Very Easy (5) |
| --- | --- | --- | --- | --- | --- |
| Hand Sanitizer (1) |  |  |  |  |  |
| Disposable Gloves (2) |  |  |  |  |  |
| Disposable Gowns (3) |  |  |  |  |  |
| Disposable Masks (4) |  |  |  |  |  |
| N95 Masks (5) |  |  |  |  |  |
| Facial Protective Shields (6) |  |  |  |  |  |
| Telemedicine Capacities (7) |  |  |  |  |  |

Display This Question:

If Have you heard about COVID-19 (Coronavirus Disease 2019)? = Yes

Q25 Please mark how easy or difficult it would be to obtain a COVID-19 test for people in the following situations.

|  | Very Hard (1) | Somewhat hard (2) | Not Sure (3) | Somewhat Easy (4) | Very Easy (5) |
| --- | --- | --- | --- | --- | --- |
| A patient who is exhibiting severe COVID-19 symptoms (1) |  |  |  |  |  |
| A patient who is suspected of having COVID-19 due to exposure history, but is not yet exhibiting severe symptoms (2) |  |  |  |  |  |
| You or a fellow healthcare professional who are suspected of having COVID-19 but are not yet exhibiting severe symptoms (3) |  |  |  |  |  |

Display This Question:

If Have you heard about COVID-19 (Coronavirus Disease 2019)? = Yes

Q26 Please mark how easy or difficult it would be for people in the following situations to access adequate treatment resources and healthcare facilities.

|  | Very Hard (1) | Somewhat hard (2) | Not Sure (3) | Somewhat Easy (4) | Very Easy (5) |
| --- | --- | --- | --- | --- | --- |
| A patient exhibiting severe COVID-19 symptoms without a confirmed COVID-19 diagnosis (1) |  |  |  |  |  |
| A patient with a confirmed COVID-19 diagnosis (2) |  |  |  |  |  |
| You or a fellow healthcare professional with a confirmed COVID-19 diagnosis (3) |  |  |  |  |  |

Display This Question:

If Have you heard about COVID-19 (Coronavirus Disease 2019)? = Yes

Q27 Please mark how much you agree with the following statements.

|  | Strongly Disagree (1) | Disagree (2) | Not Sure (3) | Agree (4) | Strongly agree (5) |
| --- | --- | --- | --- | --- | --- |
| The Somali lockdown has been effective in reducing cases and transmission of COVID-19 (1) |  |  |  |  |  |
| The Somali lockdown has made it easier for COVID-19 patients to received continued treatment (2) |  |  |  |  |  |
| The Somali lockdown has made it easier for patients without COVID-19 and with pre-existing conditions to receive continued treatment (3) |  |  |  |  |  |

Display This Question:

If Have you heard about COVID-19 (Coronavirus Disease 2019)? = Yes

Q28 Please mark how much you agree with the following statements.

|  | Strongly Disagree (1) | Disagree (2) | Not Sure (3) | Agree (4) | Strongly agree (5) |
| --- | --- | --- | --- | --- | --- |
| I am aware of Somalia's public health response to the COVID-19 outbreak (1) |  |  |  |  |  |
| The Somali government is doing enough to protect its residents from an emerging COVID-19 outbreak (2) |  |  |  |  |  |
| The Somali government is doing enough to protect its health care professionals from an emerging COVID-19 outbreak (3) |  |  |  |  |  |
| Somalia is in a good position to contain an emerging COVID-19 outbreak (4) |  |  |  |  |  |

Display This Question:

If Have you heard about COVID-19 (Coronavirus Disease 2019)? = Yes

Q29 The following are U.S. CDC recommendations for the formation of an effective, national pandemic response. To what degree do you believe that the Somali response is resulting in the successful execution of each of these recommendations? (1 = "Not at all" and 5 = "Excellently")

| Develop or revise a national pandemic response plan (1) | 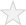 | 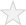 | 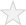 | 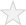 | 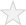 |
| --- | --- | --- | --- | --- | --- |
| Ensure that capacity is in place to identify and, where required, transport travelers with suspected pandemic COVID-19 infection to appropriate medical facilities (2) | 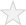 | 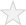 | 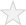 | 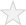 | 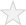 |
| Establish facilities to safely assess and isolate those with COVID-19 symptoms (3) | 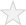 | 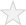 | 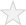 | 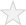 | 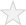 |
| Establish surveillance systems and capacities to monitor and characterize COVID-19 activity, linking epidemiological and virological information (4) | 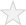 | 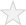 | 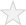 | 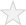 | 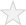 |
| Develop clinical management guidelines for patients with suspected or confirmed infection, addressing location of treatment, triage, treatment protocols, laboratory testing, and specimen collection (5) | 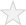 | 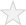 | 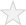 | 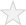 | 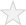 |
| Establish a central authority to oversee continuity of essential services during the pandemic (6) | 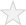 | 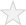 | 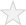 | 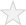 | 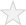 |
| Develop sufficient laboratory capacity to test subjects in potential hotspots (7) | 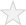 | 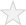 | 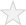 | 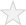 | 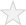 |

Display This Question:

If Have you heard about COVID-19 (Coronavirus Disease 2019)? = Yes

Q30 Please comment on what you believe the biggest gaps in the Somali response to COVID-19 and any recommendations you may have to address those gaps.

________________________________________________________________

________________________________________________________________

________________________________________________________________

________________________________________________________________

________________________________________________________________

Display This Question:

If Have you heard about COVID-19 (Coronavirus Disease 2019)? = Yes

Q31 Do you have any additional information you would like to add?

________________________________________________________________

________________________________________________________________

________________________________________________________________

________________________________________________________________

________________________________________________________________

End of Block: Experience + Attitudes
