# Supplementary material for: Knowledge of and preparedness for COVID-19 among Somali healthcare professionals: A cross-sectional study
Source: PLoS One. 2021 Nov 23;16(11):e0259981. doi: 10.1371/journal.pone.0259981 (PMC8610262; doi:10.1371/journal.pone.0259981)
Supplement: Attachment — Submitted filename: Response to Reviewers_2nd.docx [file pone.0259981.s004.docx]

October 6^th^, 2021

Dear Editor,

Thank you very much for your review of our manuscript for *PLOS One* entitled, "Knowledge of and preparedness for COVID-19 among Somali healthcare professionals: a cross-sectional study." We appreciate the editors’ and reviewers’ time and believe the edits have strengthened the manuscript considerably. We have addressed the editors’ and reviewers’ feedback below and have submitted a revised manuscript with tracked changes that correspond to the edits outlined in this letter.

I hope that we have addressed all the concerns and that the manuscript is now suitable for publication. Please do not hesitate to contact me if you have any questions or comments.

Sincerely,

Kaveh Khoshnood, PhD, MPH

**Response to Reviewers for “Knowledge of and preparedness for COVID-19 among Somali healthcare professionals: a cross-sectional study”**

**Reviewer comments:**

**Comment 1:** I would suggest that the authors include their response to my previous query, or at least a part of it, in the Study limitations section, so that it is also available to the readers: Response 13.1.

**Response 1:** Thank you for this important suggestion. We have incorporated our response (Response 13.1) to your previous query in our Limitations section.

The section now reads:

*This study is subject to various limitations. First, this study utilized a convenience sample, which is likely not representative of the entire Somali healthcare workforce. In addition, the sampling method’s reliance on connections to the Hagarla Institute may produce a sampling bias geared towards healthcare workers located closer to Mogadishu. These biases may be partially responsible for our sample’s younger age profile. Similarly, given challenges associated with data collection in the field, especially during a COVID-19 wave, this study did not collect information relating to non-respondents. As such, this may also bias the generalizability of the study findings. Because our multivariate analysis did not adjust for potential confounding factors, residual confounding may also have been introduced.* ***Furthermore, as this study utilized a cross-sectional design, it cannot be used to analyze the evolution of COVID-19 knowledge, access to personal protective equipment, and perceptions of the Somali national response over an extended period of time. This study took place during Somalia’s first wave of COVID-19. Since then, larger waves have been recorded, which is expected to have impacted knowledge of and preparedness for COVID-19 among Somali healthcare professionals. Although this study does not capture the progression of Somalia’s COVID-19 situation, the study findings provide valuable insights into pertinent factors that must be considered in preparing for and choosing how to disseminate information during future outbreaks. These findings also have direct implications for the development of future research priorities and interventions related to COVID-19 in Somalia, as well as other similar resource-constrained settings. This is a context where very little methodologically rigorous research has been conducted, and we are hopeful that this study will serve as a reference point for future public health and research initiatives being developed currently and beyond the COVID-19 pandemic.***

**Comment 2:** Sample and setting: The following information has been added: “If these criteria were met, a brief presentation of the purpose, procedure, and requirements for participation were given privately.” However, it is still not clear to whom this presentation was given? How were these participants identified and chosen? From a specific hospital? Hospital network? Professional society? From social media? A hint on this is given in the lines below, but it is still not clear: “recruited potential participants within healthcare-delivering institutions in and around Mogadishu, Somalia.”

**Response 2:** Thank you for bringing this point to our attention. We recognize the need for further detail as to how participants were identified and have added further information in the Sample and Setting subsection of the Methods section.

The subsection now reads:

*A convenience sample of 364 healthcare workers in Somalia was obtained between June and August 2020. Eligibility requirements for participation in this study included being 18 years or older, physically able to complete the survey, willing to take part in the study, and working within a healthcare profession. If these criteria were met, a brief presentation of the purpose, procedure, and requirements for participation were given privately. Participants were told that they could withdraw from the study at any time and that their participation was entirely voluntary. Given the setting, population of interest, eligibility requirements, and the limited risks associated with participation, verbal, as opposed to written, consent was obtained from participants to facilitate timely recruitment.* ***Verbal, informed consent was witnessed and documented for each participant by trained staff from the Hagarla Institute, a non-profit organization dedicated to furthering clinical research, capacity-building, and skills transfer for medical personnel across Africa. Hagarla Institute personnel visited healthcare-delivering institutions within their network in and around Mogadishu and identified and recruited healthcare professionals who satisfied the aforementioned eligibility requirements. After receiving consent from participants, Hagarla Institute staff interview-administered each survey.*** *All responses were anonymous and kept confidential. This study received approval from the ethics board at SIMAD University in Somalia and was deemed exempt from review by the Yale Human Subjects Committee (ID #2000028344).*
