# Supplementary material for: Knowledge of and preparedness for COVID-19 among Somali healthcare professionals: A cross-sectional study
Source: PLoS One. 2021 Nov 23;16(11):e0259981. doi: 10.1371/journal.pone.0259981 (PMC8610262; doi:10.1371/journal.pone.0259981)
Supplement: Attachment — Submitted filename: Response to Reviewers_3rd.docx [file pone.0259981.s005.docx]

October 15^th^, 2021

Dear Editor,

Thank you very much for your review of our manuscript for *PLOS One* entitled, "Knowledge of and preparedness for COVID-19 among Somali healthcare professionals: a cross-sectional study." We appreciate the editors’ and reviewers’ time and believe the edits have strengthened the manuscript considerably. We have addressed the editors’ and reviewers’ feedback below and have submitted a revised manuscript with tracked changes that correspond to the edits outlined in this letter.

I hope that we have addressed all the concerns and that the manuscript is now suitable for publication. Please do not hesitate to contact me if you have any questions or comments.

Sincerely,

Kaveh Khoshnood, PhD, MPH

**Response to Reviewers for “Knowledge of and preparedness for COVID-19 among Somali healthcare professionals: a cross-sectional study”**

**Reviewer comments:**

**Comment 1:** Unfortunately, the authors have not addressed my previous comment:
“Sample and setting: The following information has been added: “If these criteria were met, a brief presentation of the purpose, procedure, and requirements for participation were given privately.” However, it is still not clear to whom this presentation was given? How were these participants identified and chosen? From a specific hospital? Hospital network? Professional society? From social media? A hint on this is given in the lines below, but it is still not clear: “recruited potential participants within healthcare-delivering institutions in and around Mogadishu, Somalia””

I would suggest that the authors briefly explain how they identified potential participants. For example, did they use an existing database of medical personnel from a certain hospital or a certain profession society? Or were participants approached in their own hospital during their working hours? Also, this section should clarify through which means they reached out to study participants, by phone, email, in person, etc.

**Response 1:** Thank you very much for this important suggestion. We recognize the need for further detail as to how potential participants were identified. As such, we had added more information to clarify that participants were reached out to in-person and that participants were approached in their own hospital during their working hours. Trained staff from the Hagarla Institute visited health facilities within their network and approached potential participants in-person during their working hours to assess eligibility and willingness to participate.

The subsection now reads:

*“A convenience sample of 364 healthcare workers in Somalia was obtained between June and August 2020. Eligibility requirements for participation in this study included being 18 years or older, physically able to complete the survey, willing to take part in the study, and working within a healthcare profession.* ***Trained staff from the Hagarla Institute, a non-profit organization dedicated to furthering clinical research, capacity-building, and skills transfer for medical personnel across Africa, visited healthcare-delivering institutions within their network in and around Mogadishu and thereafter identified and recruited healthcare professionals who satisfied the aforementioned eligibility requirements. Within each hospital or clinic, Hagarla Institute staff approached potential participants in-person during their working hours to assess eligibility and willingness to participate.*** *If eligibility criteria were met, a brief presentation of the purpose, procedure, and requirements for participation were given privately. Participants were told that they could withdraw from the study at any time and that their participation was entirely voluntary. Given the setting, population of interest, eligibility requirements, and the limited risks associated with participation, verbal, as opposed to written, consent was obtained from participants to facilitate timely recruitment. Verbal, informed consent was witnessed and documented for each participant by Hagarla Institute staff. After receiving consent from participants, Hagarla Institute staff interview-administered each survey. All responses were anonymous and kept confidential. This study received approval from the ethics board at SIMAD University in Somalia and was deemed exempt from review by the Yale Human Subjects Committee (ID #2000028344).”*
